# Understanding biogeographical patterns in the western Balkan Peninsula using environmental niche modelling and geostatistics in polymorphic *Edraianthus tenuifolius*

**DOI:** 10.1093/aobpla/ply064

**Published:** 2018-10-11

**Authors:** Peter Glasnović, Martina Temunović, Dmitar Lakušić, Tamara Rakić, Valentina Brečko Grubar, Boštjan Surina

**Affiliations:** 1 Faculty of Mathematics, Natural Sciences and Information Technologies, University of Primorska, Glagoljaška, Koper, Slovenia; 2 Faculty of Forestry, Department of Forest Genetics, Dendrology and Botany, University of Zagreb, Svetošimunska, Zagreb, Croatia; 3 Faculty of Biology, Institute of Botany and Botanical Garden ‘Jevremovac’, University of Belgrade, Takovska, Belgrade, Serbia; 4 Faculty of Humanities, University of Primorska, Titov trg, Koper, Slovenia; 5 Natural History Museum Rijeka, Lorenzov Prolaz, Rijeka, Croatia

**Keywords:** Balkan refugia, *Edraianthus tenuifolius*, environmental niche modelling, geostatistics, last glacial maximum, morphological variability, ‘refugia within refugia’ model

## Abstract

The Balkan Peninsula represents one of the three southern European glacial refugia where biodiversity persisted throughout the climatically unstable Quaternary. This study considered the ‘refugia within refugia’ model, which assumes the environmental heterogeneity over time and space in larger refugia. To better understand patterns shaped during the Quaternary climatic oscillations, the present and last glacial maximum (LGM) environmental conditions and current morphological variability of *Edraianthus tenuifolius*, an endemic plant of the western Balkans with a well-known therphical structure, were considered. Potential present and LGM distributions were studied through environmental niche modelling using 161 data of occurrences and six bioclimatic variables, hindcasting the model to LGM conditions using three different global circulation models. To test the geographical variability of the reproductive region, 41 characters of 667 inflorescences from 35 populations within the distribution range were measured. Geographical patterns, using geostatistics together with univariate and multivariate statistical approaches, were analysed. The environmental niche model suggested the current potential distribution in correspondence to its known occurrences. The hindcast to LGM conditions suggested two separate areas of environmental suitability, one in the present-day northern Adriatic coast of Croatia (Istrian Peninsula, Kvarner) and another on the present-day south-eastern Adriatic coast (southern Dalmatia, Montenegro and northern Albania). Morphological variability showed a similar pattern, where southern populations separated from northern populations according to a major split in the central part of its distribution range (central Dalmatia). On other levels, stronger barriers were predicted to separate northern populations from the eastern Istrian Peninsula and the Kvarner area. The results suggested congruent biogeographical patterns to the already known phylogeographical structure. Both environmental niche modelling and the geographical variability of morphological characters suggested spatial partitioning, indicating the potential presence of two separate refugia during the LGM.

## Teaser

Combining occurrence data with present and LGM environmental conditions coupled with spatial analysis of phenotypic plasticity of floral characters the authors reveal once again the intricate biogeographic patterns within the Balkan Peninsula. The endemic plant Edraianthus tenuifolius shows patterns of geographical variation that strongly support the 'refugia within refugia' concept in the South European refugia. Moreover, results from the present work are congruent with a previous phylogeographical study supporting the recognition of refugial areas for thermophytic plant taxa during the glacial events of the Pleistocene.

## Introduction

Identifying Quaternary glacial refugia has been a major issue in biogeographical research efforts over the last century (Feliner 2011). The concept of refugia presumes a contraction in species range to a particular geographic area along with a reduction in its abundance due to decreased and/or unfavourable environmental conditions (Bennett and Provan 2008). Traditionally, such areas were identified according to the distribution of vicariant taxa, disjunctions within continuous species distributions or by studying fossils, macroscopic plant remains and pollen deposits (e.g. Wettstein 1898; Huntley and Birks 1983; Carrión *et al.* 2003; Waltari *et al.* 2007; Brus 2010). Throughout the last decades, however, phylogeography has become the dominant approach in understanding biogeographical patterns and recognizing areas that served as potential glacial refugia (Feliner 2011). With the development of molecular methods and their implementation into biogeographical studies, the role of the southern European peninsulas—the Balkan, the Apennine and the Iberian Peninsulas—as refugia for temperate biota throughout the Quaternary glaciation periods was stressed (Taberlet *et al.* 1998; Hewitt 1999, 2000). Early phylogeographical studies on European plants focussed on tree species, testing how singular taxa recolonized northern regions during warmer periods from the three main southern glacial refugia (Lagercrantz and Ryman 1990; Konnert and Bergmann 1995; Demesure *et al.* 1996; Dumolin-Lapègue *et al.* 1997; Hampe *et al.* 2003), or alternatively, from their northernmost range limits (Petit *et al.* 2003; Magri *et al.* 2006). By gaining new information about the phylogeographical patterns of Iberian taxa, it became evident that many lineages show strong genetic subdivisions indicating population isolation in separate, more restricted areas, introducing a new concept of ‘refugia within refugia’ (Gomez and Lunt 2007). This concept has been confirmed as a pattern throughout a number of molecular studies about the diversity of European biota, leading to the recognition of numerous additional areas that may have acted as more restricted refugia within the three southern European Peninsulas. For example, based on the existence of phylogeographical patterns of Alpine plants and regional geomorphological characteristics, potential refugia have been recognized in the Alps (Schönswetter *et al.* 2005). Additionally, a detailed map of areas potentially acting as plant refugia within the wider Mediterranean basin has been synthesized by Médail and Diadema (2009).

The Balkan Peninsula, largely overlooked over the last century, has obtained much attention from the international botanical community during the last decades. Particular consideration has been given to the molecular systematics, phylogeny and phylogeography of endemic taxa or taxa with the centre of diversity in the Balkans (e.g. *Edraianthus*: Stefanović *et al.* 2008; Surina *et al.* 2011, 2014*b*). While studies based on molecular data increased, traditional approaches such as spatial analysis of morphological plant characters remained largely neglected. Only recently morphology combined with molecular phylogenetics was used to underpin the highly disjunct distribution of *Wulfenia* between the southeastern Alps, southern Dinaric Alps and the Amanos Mts. (Surina *et al.* 2014*a*), to understand the systematic relationship within the genus *Amphoricarpos* from the central and southern Dinaric Alps (Caković *et al.* 2015) and to clarify the relations within *Campanula pyramidalis* (Janković *et al.* 2016) and *Alyssum montanum–A. repens* species complexes in the Balkans (Španiel *et al.* 2017*a*, *b*). However, studies that link patterns of Balkan plant diversity to ecological processes are yet to be conducted.

In addition to phylogeography, environmental niche modelling (ENM; also known as ‘habitat suitability’ or ‘species distribution’ modelling) serves as a powerful tool to detect areas of environmental suitability that could have acted as refugia (Guisan and Zimmermann 2000; Waltari *et al.* 2007). By combining known species distributions with environmental (bioclimatic) variables through the use of appropriate modelling algorithms, one can project current conditions onto paleoclimatic data (hindcasting). This enables us to infer their paleodistributions and past range dynamics as well as to detect their putative refugial areas. Niche modelling was used to understand past tree dynamics on the Iberian Peninsula (Rodríguez-Sanchéz *et al.* 2010) and to elucidate genetic patterns of the plants of the Balkan Peninsula (Surina *et al.* 2014*b*; Rešetnik *et al.* 2016).

The present study focuses on *Edraianthus tenuifolius*, a suitable model organism to study biogeographical patterns on the Balkan Peninsula. The plant is endemic to the Balkans and has a well-known phylogeographical pattern. First, our aim was to furtherly inspect the biogeographical processes by combining the known species distribution with the current and Last Glacial Maximum (LGM) environmental (bioclimatic) conditions. This allowed us to better understand the biogeographical patterns and possibly identify past areas of environmental suitability within the Balkan refugia. Second, by examining morphological characteristics of floral traits and implementing them into GIS and spatial statistics, our aim was also to understand how past biogeographical inheritances are reflected in patterns of current morphological variability through the distribution range of the plant.

## Methods

### Study species

**Figure 1.**
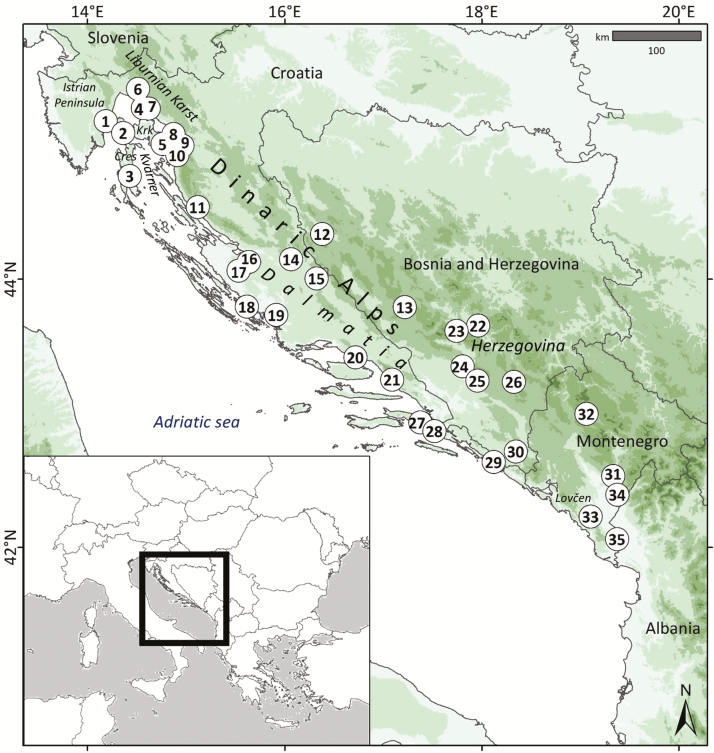
Studied area: eastern Adriatic coast and the Dinaric Alps within the western part of the Balkan Peninsula. Numbers correspond to sampled populations of *E. tenuifolius*. Detailed information available in **Supporting Information—Table S1**.

*Edraianthus tenuifolius* (Waldstein and Kitaibel) A.DC. (Campanulaceae) is an endemic plant of the western Balkan Peninsula (Fig. 1), inhabiting rocky (sub)Mediterranean grasslands from the Istrian Peninsula (Slovenia and Croatia) to northern Albania.

**Figure 2.**
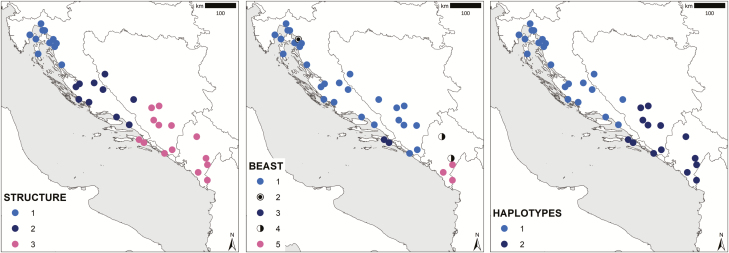
Visualization of subdivisions of *E. tenuifolius* populations considered in the present study into groups based on different methods: STRUCTURE, haplotype analysis with BEAST and diversity of HAPLOTYPES according to Surina *et al.* 2011.

Distributed predominantly in coastal areas along the eastern Adriatic Sea, it can be found from sea level up to 1500 m. Several populations are thriving deep in the mainland along river valleys that are influenced by the Mediterranean climate. Early research has already highlighted evident morphological variability within this taxon. Janchen (1910) reported on glabrous specimens occurring in the northern part of its distribution range, which he described as a special form *E. tenuifolius* f. *semiglabra*. In addition, Lakušić (1974) reported on polymorphism in morphological characters in his monography on the genus *Edraianthus*. According to the author, the typical form is characteristic only of the Mediterranean and (sub)Mediterranean rocky grasslands of the coastal area. He observed much more robust and less hairy specimens with a higher number of flowers in their inflorescence (*E. tenuifolius* f. *gigantea*) in the dolomitic area of central Herzegovina. In line with Janchen (1910), he reported distinctive, mesophytic forms (*E. tenuifolius* f. *glabra*) from the northern part of the distribution range. In her PhD thesis, Rakić (2010) highlighted pronounced morphological and anatomical differentiation between populations of *E. tenuifolius* from Montenegro, where a hybrid between *E. tenuifolius* and *E. wettsteinii* subsp. *lovcenicus* was described (Mt. Lovčen: Lakušić *et al.* 2009), pointing out the importance of reticulate evolution in the diversification of the genus *Edraianthus*. More recently, *E. tenuifolius* has been considered a good model organism to interpret the presence of phylogeographical patterns in the Balkans (Surina *et al.* 2011). In this analysis, based on AFLP and plastid DNA genetic markers, different statistical approaches resulted in different interpretations of the spatial genetic structure of *E. tenuifolius*. As a result, populations can be divided into several groups: five groups (Beast analysis), three groups (Structure analysis) or only two groups (Haplotypes; Fig. 2 and **see ****Supporting Information—Table S1**). In the last case, the northern group is characterized by a single haplotype, whereas the southern group is extremely diverse in terms of different haplotypes.

### Environmental niche modelling

A database of 161 occurrences from various sources including field work, literature, herbaria collections and public databases was created. All collected data were precisely georeferenced using a GPS or appropriate cartography combined with GIS tools (ESRI ArcGIS, ver. 10.1.) in the WGS84 coordinate system.

The environmental space was determined according to a set of six bioclimatic variables selected based on the procedure described subsequently. Current data variables with resolutions of 30 arc-seconds available from the Worldclim web site (Hijmans *et al.* 2005) were employed. For the evaluation of the environmental space during the LGM period (approximately 22 000 years ago), the same set of variables was used at the resolution of 2.5 arc-minutes, calibrated according to three different global circulation models (GCMs): MIROC—Model for Interdisciplinary Research on Climate (Hasumi and Emori 2004), CCSM4—The Community Climate System Model Version 4 (Gent *et al.* 2011) and MPI-ESM-P—Max–Planck-Institute Earth System Model (Giorgetta *et al.* 2013) were used. Variables were not aggregated to the same resolution. To select bioclimatic variables for ENM, we first computed Pearson’s correlation coefficients (*r*) between all available variables, in order to eliminate highly correlated and thus redundant environmental variables for the explanation of species presence. Following this procedure, different sets of variables with |*r*| < 0.8 were retained and used to build the models. The final model and the corresponding variable set were selected based on the model performance evaluation (described in what follows). The environmental background was limited to the geographic area covered by the Dinaric Alps and the eastern Adriatic coastal area, including islands.

Environmental niche modelling was performed using Maxent (v. 3.3.3k), an algorithm to identify species’ suitable environmental space from incomplete information of occurrence (Phillips *et al.* 2006). To avoid overfitting the input data, the final model was chosen based on the appropriate value of regularization multipliers. Presence data and pseudo-absence, using 10 000 background points, were compared in order to identify areas of environmental suitability (Barbet-Massin *et al.* 2012). Twenty replicates for each model with cross-validation were performed. To understand the contribution of environmental variables to each of the built models, the per cent contribution and jack-knife tests were taken into consideration. The model performance was assessed based on two criteria: the area under the receiver operating characteristic curve (AUC) and the Akaike Information Criteria (AIC) calculated in the ENM Tools (Warren *et al.* 2010). The most parsimonious model based on the highest AUC and/or lowest AIC score was selected. The final paleomodel was calculated as a consensus model across three different LGM projections as the average pixel value using the ‘raster calculator’ in ESRI ArcGIS ver. 10.1. All processing and visualizations of Maxent results were performed in ESRI ArcGIS 10.1. To obtain a presence/absence map, the resulting Maxent continuous habitat suitability map was transformed into a binary map using the minimum training presence (MTP) logistic threshold, which represents the lowest suitability value for an observed presence record and has no omission of the known training occurrence points (Liu *et al.* 2005). It can be interpreted as areas at least as environmentally suitable as those where the species has actually been recorded (Pearson *et al.* 2007).

In addition, we evaluated how climatic conditions during the LGM differed between areas recognized as suitable and those recognized as environmentally unsuitable. The analysis was carried out by comparing the modelling results based on six selected bioclimatic variables describing the overall climate: BIO1—annual mean temperature; BIO5—maximal temperature of the warmest month; BIO6—minimal temperature of the coldest month; BIO12—annual precipitation; BIO16—precipitation of the wettest quarter and BIO 17—precipitation of the driest quarter. First, the values of the selected variables in the areas recognized as suitable and unsuitable according to Maxent projections of LGM environmental conditions were extracted. This was done using the ‘extract multi values to point tool’ in ESRI ArcGIS 10.1. The values for each environmental variable between the analysed areas were then compared based on parametric or non-parametric approaches using the IBM SPSS Statistics 21 software.

### Spatial patterns of morphological variability

To obtain a multivariate matrix of floral characters, 667 inflorescences (approximately 20 per location) from 35 populations of *E. tenuifolius* (Fig. 1 and **Supporting Information—Table S1**) were collected within its distribution range and in accordance with populations that were considered in the phylogeographic analysis (Surina *et al.* 2011).

Only characters on the reproductive parts (inflorescence) were taken into consideration. Floral traits in *Lobelia siphilitica*, a member of Campanulaceae family from North America, showed less plasticity as a response to changing environmental conditions in comparison with the vegetative characters (Caruso 2006). Moreover, studies examining the vegetative traits of the closely related *E. graminifolius* showed that these are too diverse and lacking any spatial pattern (Rakić *et al.* 2012). Inflorescences were stored *in situ* in an admixture of glycerol and ethanol (50:50, v/v). The bracts and the flowers were subsequently dissected and photographed in the laboratory. A total of 41 morphological characters of each inflorescence [**see ****Supporting Information—Table S5**] were measured using the program Digimizer. In preliminary inspections, differences in the floral size within the inflorescence were observed; hence all measurements were conducted on the most central flower (first to fully develop) and on the largest lateral flower in the inflorescence.

Voucher specimens from each sampled population were stored in the herbaria of the University of Primorska (Koper, Slovenia), the Natural History Museum of Rijeka (Rijeka, Croatia; NHMR) and the University of Belgrade (Belgrade, Serbia; BEOU).

To provide continuous rasters of morphological characters, the average values of character measurements of each population were interpolated using the kriging interpolation method within the area of environmental suitability obtained with Maxent modelling. Accurate kriging predictions could have a mean error close to 0, the smallest possible root-mean-square error and average standard error, and root-mean-square standardized error close to 1. A correct prediction would have mean average standard errors close to the root mean squared prediction errors.

The obtained rasters were reclassified into equal intervals within minimum (1) and maximum (100) values using the ‘raster calculator’ and joined those in a singular file by employing the ‘composite band tool’. This file was then used to perform the spatial principal component analysis (sPCA) using the ‘principal component analysis tool’, which allowed us to recognize multivariate gradients in space and areas of higher morphological variability (Brito *et al.* 2008; Tomović *et al.* 2010; Martinez-Freira and Brito 2013). Based on the sPCA results, the axes that cumulatively explain at least 90 % of the variability were isolated using the ‘make raster layer tool’. By using the ‘iso cluster tool’, clusters of multivariate data recognized in the sPCA were identified. The number of clusters was selected based on the published results of genetic spatial patterns (Surina *et al.* 2011). Clusters were calculated using the ‘dendrogram tool’ and their values were assigned through the ‘maximum likelihood classification tool’. All the analyses and visualizations of the results were performed in ArcGIS 10.1.

To recognize geographical breaks of multivariate variability and their respective ranks, a distance matrix of multivariate morphological variability and the metric distance matrix were compared. Both matrices were constructed with the ‘vegdist tool’ in the vegan R package (Oksanen *et al.* 2018). The distance matrix of multivariate morphological variability was constructed calculating the Gower distance. The obtained data were analysed using the Barrier 2.2. software (Manni *et al.* 2004) that employs the Monmonier algorithm which compares two or more distance matrices and seeks possible geographic breaks. The results of the Barrier analysis were visualized using ESRI ArcGIS 10.1.

### Congruencies between morphological variability and biogeographical patterns

To verify whether the morphological variability corresponds to the already known spatial patterns, the studied populations were assessed based on different genetic groups that had been identified in the phylogeographic analysis (Fig. 2 and **see ****Supporting Information—Table S1**) (Surina *et al.* 2011). Moreover, morphological variability was tested also on groups identified with Iso Cluster and Barrier [**see ****Supporting Information—Fig. S1**]. Since not all characters were available for all the populations, the present analysis includes only 29 characters measured on the bracts and on the central flower of the inflorescence available for all the populations. The preliminary analysis showed that central flowers differ from subsequently developed lateral flowers by a significantly (*P* < 0.05) higher total corolla height.

The initial step was to test whether there were statistically significant differences (*P* < 0.05) in characters between populations grouped based on different methods. Using the Kolmogorov–Smirnov test, the normal distribution of the input data was verified. The parametric tests (ANOVA and *post hoc* test) were used when these data met the criterion of normal distribution and equal variance; otherwise, the Kruskal–Wallis test was applied. Differences between groups were statistically tested on 29 characters of samples represented in the different groups. Due to the very large amount of the output data, the results were expressed as a per cent of statistically significant differences within and between groups.

In addition, the significance of the groups was assessed by the classification method of the canonical discriminant analysis (CDA), where only normally distributed and less inter-correlated (|*r*|< 0.8) characters were included in the analysis. All statistical analyses were performed in the IBM SPSS Statistics 21 software.

## Results

### Spatial patterns based on current and past environmental suitability

A set of six bioclimatic variables properly met statistical requirements and best explained the model results as predictors (Table 1 and **see ****Supporting Information—Table S2**).

**Table 1.** Bioclimatic variables used for modelling and their per cent contribution showed as average across runs.

Bioclimatic variable	Description	Per cent contribution, %
BIO13	Precipitation of Wettest Month	55.3
BIO9	Mean Temperature of Driest Quarter	14.6
BIO17	Precipitation of Driest Quarter	14.1
BIO7	Temperature Annual Range	8.8
BIO8	Mean Temperature of Wettest Quarter	4.0
BIO2	Mean Diurnal Range	3.1

**Figure 3.**
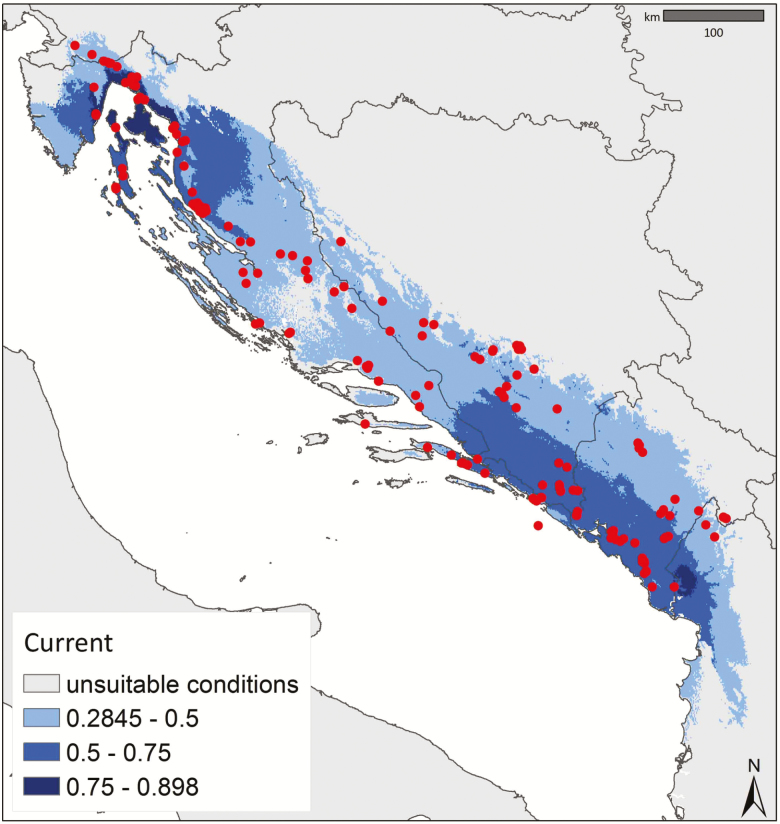
Predicted current environmental suitability for *E. tenuifolius*. Red dots correspond to the occurrences used for modelling.

The most parsimonious model was obtained when setting the regularization multiplier value at 5 based on the lowest AIC_c_ (3614.89). TheAUC_Test_ value for the model was 0.9327. The Maxent results were processed and areas of environmental suitability were visualized by setting the MTP threshold at 0.253. Cells with lower values than the threshold were considered as unsuitable conditions. Modelling considering current conditions predicted a suitable environment throughout the area matching the present distribution range of *E. tenuifolius* (Fig. 3).

**Figure 4.**
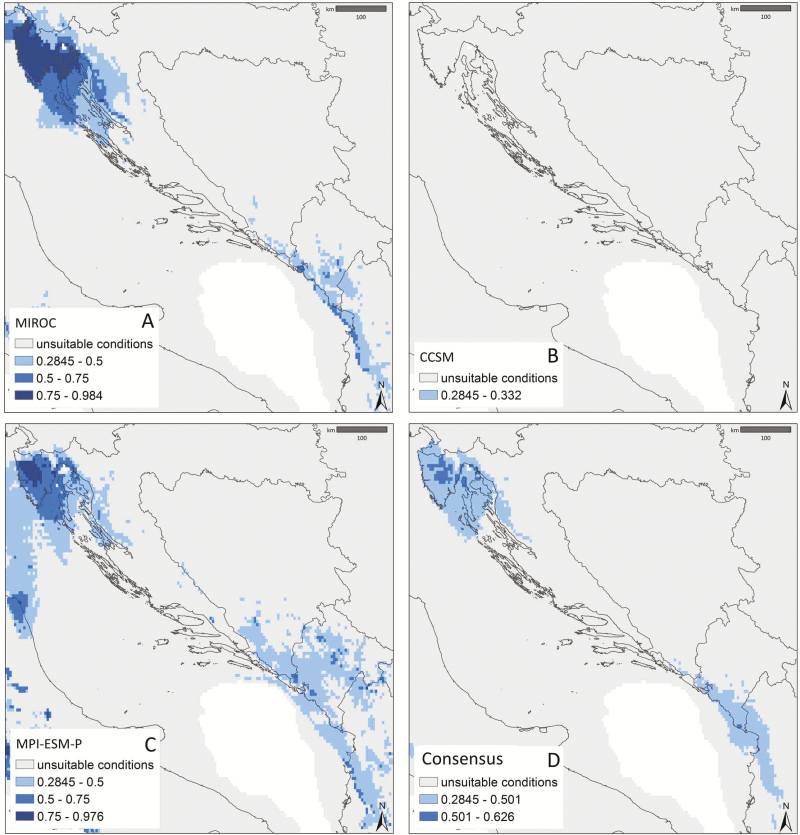
Hindcast environmental suitability for *E. tenuifolius* under LGM conditions based on different global circulation models, MIROC (A), CCSM (B), MPI-ESM-P (C) and their consensus model (D).

Models hindcast to LGM conditions supported different scenarios based on projections of current ENM to the three GCMs. The CCSM model did not support any environmentally suitable areas within current circumscription (Fig. 4B). The MIROC and MPI-ESM-P models (Fig. 4A and C) indicated three distinctive areas: (i) the northwestern (NW; Istrian Peninsula and Kvarner); (ii) the central (northern and central Dalmatia) and (iii) the southeastern area (SE; southern Dalmatia, Herzegovina, Montenegro and northern Albania) of the current eastern Adriatic coast. The NW and SE areas were identified as environmentally suitable during the LGM, whereas the central area was identified as unsuitable. The NW area was characterized by lower precipitation and temperature values compared with the SE area. However, both areas of LGM suitability significantly differed in being warmer and moister when compared with the central, unsuitable area [**see ****Supporting Information—Tables S3** and S4].

### Spatial patterns of morphological variability

**Figure 5.**
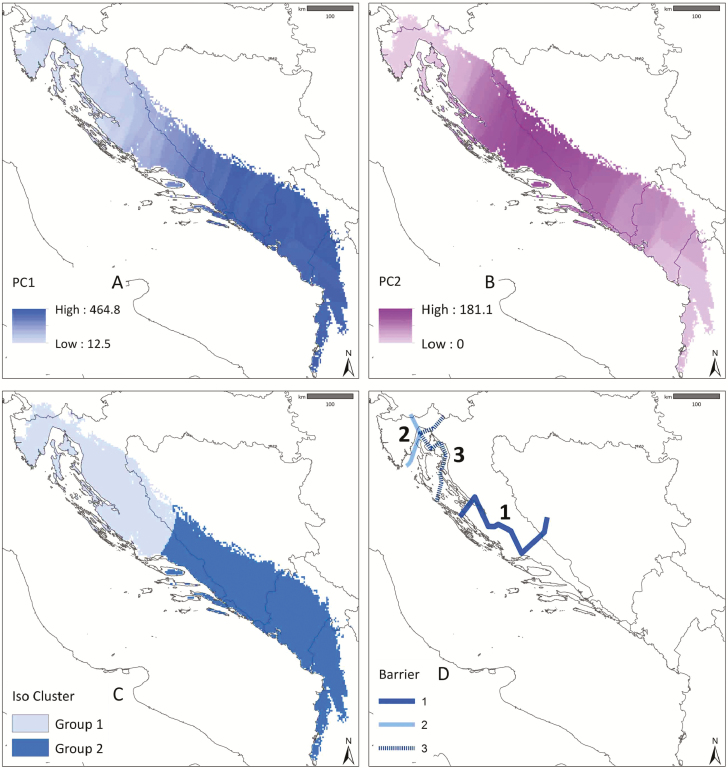
Spatial patterns of morphological variability. (A) The first and (B) second principal component (PC1 & PC2) of the spatial principal component analysis based on morphological characters of the inflorescence of *E. tenuifolius*. (C) Populations of *E. tenuifolius* classified into two groups based on maximum likelihood classification and iso cluster. (D) Visualization of the three strongest barriers obtained based on the Barrier analysis of the metric and morphological distance matrices of *E. tenuifolius*. The line width, style and labelling (1–3) reflect the strength and order of the barriers.

Using the kriging interpolation method, 41 accurate continuous raster datasets were produced [**see ****Supporting Information—Table S6**] within the distribution range of *E. tenuifolius,* which had been obtained with Maxent modelling. In the spatial principal component analysis, the first three principal components (PC) explained 96.3 % of the total variability: PC1 = 82.1 %, PC2 = 9.3 %, PC3 = 4.9 %. Cartographical visualization is presented for the first two PC axes (Fig. 5A and B). PC1 on the geographical level predicted a gradient in morphological variability from the NW part of the distribution range towards the SE, with the highest values in southern Dalmatia, Herzegovina, Montenegro and northern Albania. PC2 predicted a peak of variability in the central part of the distribution range (central and northern Dalmatia).

Two groups were proposed for clustering based on the results of the ENM and previous research (Surina *et al.* 2011). The results suggested that these two groups are separated in the central part of the species distribution range (central Dalmatia, Fig. 5C).

The analysis of distance matrices in Barrier proposed geographical breaks on different levels. The first barrier divided the NW and SE populations in central Dalmatia. The second barrier isolated the population on the Istrian Peninsula (Pop 1) from other populations, whereas the third barrier isolated the populations from the Kvarner area and the Liburnian Karst (Pops 2, 3, 5 and 6; Fig. 1 and **Supporting Information—Table S1**).

### Congruencies of recognized biogeographical patterns

The genetic groups proposed by previous studies (Surina *et al.* 2011) and groups identified in this study showed congruent results when tested with univariate (Table 2) and multivariate (Table 3) statistical approaches.

**Table 2.** Per cent distribution of statistically significant differences (*P* < 0.05) in morphological characters (*n* = 29) between different grouping methods based on ANOVA or Kruskall–Wallis test. *Post hoc* tests were applied in cases where more than two groups were identified.

Grouping method		% statistically significant differences
Structure	Group 1	78.3
	Group 2	69.6
	Group 3	87
Haplotypes (two groups)		89.7
Beast	Group 1	35.5
	Group 2	35.5
	Group 3	28.9
	Group 4	52.6
	Group 5	52.6
Isocluster (two groups)		89.7
Barrier (two groups)		89.7
Barrier	Group 1	61.9
	Group 2	49.2
	Group 3	77.8
	Group 4	77.8

**Table 3.** The percentage of *E. tenuifolius* specimens correctly classified in predefined groups based on classification approaches (Original and Cross-validated) with the canonical discriminant analysis of morphological characters.

Subdivision	Groups	Original, %	Cross-validated, %
Structure	1	84.9	84.0
	2	71.0	69.8
	3	77.6	76.1
Haplotypes	1	90.0	89.5
	2	66.5	64.5
Beast	1	96.2	95.8
	2	0.0	0.0
	3	5.0	5.0
	4	57.9	42.1
	5	43.3	41.8
Iso Cluster	1	82.9	82.1
	2	62.5	62.2
Barrier 1 (two groups)	1	73.2	72.9
	2	76.7	75.9
Barrier 3 (four groups)	1	25.0	20.0
	2	67.1	67.1
	3	60.2	58.2
	4	87.8	86.6
Barrier 3 (three northern groups)	1	85.0	75.0
	2	86.1	82.3
	3	72.4	69.9

The comparison of characters among groups was statistically well supported in cases where the division of populations proposed two groups (Haplotypes, Iso clusters and first level Barrier; Table 2). In all three cases, 26 out of 29 (89.7 %) characters exhibited statistically significant differences. In the cases when groups were defined based on Barrier and Structure, all (29) characters expressed statistically significant differences when ANOVA or Kruskall–Wallis tests were applied. However, the results of *post hoc* tests within the four geographic partitions proposed by Barrier (three levels) showed 61.9 % (Group 1), 49.2 % (Group 2), 77.8 % (Group 3) and 77.8 % (Group 4) statistically significant differences. In the case of groups employing the Structure analysis, the three groups resulted in 78.3 % (Group 1), 69.6 % (Group 2) and 87.0 % (Group 3) statistically significant differences. The Barrier group populations isolated to the northern Adriatic islands of Cres and Krk (Pops 2, 3 and 5) and one nearby mainland population on the Liburnian Karst (Pop 6; Fig. 1 and **Supporting Information—Table S1**) significantly differed from the rest of the groups based on all investigated characters. The uniqueness of these populations with small inflorescences, lower number of flowers and bracts, was clearly pointed out by descriptive data and their cartographical representation. An overall tendency showed that the differences between groups decreased with increasing numbers of groups. The groups defined as a result of the Beast analysis showed the lowest rate of statistically significant differences (Group 1—35.5 %, Group 2—35.5 %, Group 3—28.9 %, Group 4—52.6 %, Group 5—52.6 %).

The CDA did not support complete segregation of the groups. However, very informative results were obtained when testing the classification based on two groups obtained by Haplotype, Iso Cluster and Barrier analyses (Table 2). These results supported the scenario that these groups that are separated in central Dalmatia represent the most likely biogeographical pattern of morphological variability in *E. tenuifolius*. The correct classification of our samples decreased with the increasing number of subdivisions. However, when isolating and testing only the NW groups based on the Barrier test (Groups 1, 2 and 3), one could once again observe the distinctiveness of populations from the northern Adriatic area (Table 3).

## Discussion

### Looking for refugia within refugia

Geographic information systems and spatial modelling have become fundamental tools in studying biogeographical patterns of organism distributions. Some patterns of Balkan biodiversity were clarified through the use of these tools. For example, today the diversity of the horn viper (*Vipera ammodytes*) is better understood due to the implementation of traditional morphometric techniques into geostatistical methods and environmental niche modelling (Tomović *et al.* 2010). Niche modelling also helped to clarify the genetic structure of narrow-leafed ash (*Fraxinus angustifolia*) populations in Croatia (Temunović *et al.* 2012). In the case of *E. tenuifolius*, modelling results suggested that the predicted present distribution corresponds well with the actual known occurrences of the species. On the other hand, predicted climatic suitability during LGM conditions indicated contractions of the occurrence to the environmentally adequate areas located in today’s region of the Istrian Peninsula and Kvarner (NW part of the eastern Adriatic coast) as well as in southern Dalmatia, Montenegro and northern Albania (SE part of the eastern Adriatic coast). Several studies have emphasized the need for scepticism when interpreting results of hindcasts to past conditions due to the extrapolated nature of environmental data in geographically highly heterogeneous areas (Médail and Diadema 2009; Davis *et al.* 2014). However, our data matched interpretations of other studies addressing similar questions. Signals in the genetic structure of *E. tenuifolius* suggested a clear importance of the SE Adriatic region as an area of its continuous presence during the Quaternary glaciations (Surina *et al.* 2011). Similarly, the SE Adriatic coast appeared to have acted as a centre of diversification during the colder periods for a number of other plant taxa (*Cardamine acris*: Perný *et al.* 2004; *Cardamine maritima*: Kučera *et al.* 2008; *Campanula pyramidalis*: Lakušić *et al.* 2013; *Tanacetum cinerariifolium*: Grdiša *et al.* 2014). This area met favourable conditions probably due to the prolonged vicinity to the sea, which shrunk much further to the south during the glacial periods and significantly mitigated the climate in the southern Adriatic basin (Tzedakis 2004; Lakušić *et al.* 2013). Even if the genetic diversity in more northern populations of *E. tenuifolius* proved to be depauperated and rare alleles less common (Surina *et al.* 2011), the overall genetic structure and the present distribution of haplotypes indicated that NW regions of the eastern Adriatic coast could also have served as an important area of diversification. These data were congruent with the genetic structure of another Mediterranean plant, *T. cinerariifolium*, which also showed distinct and unique genetic patterns in the NW Adriatic region (Grdiša *et al.* 2014). A very similar biogeographical pattern is reflected in the distribution of some pairs of geographical vicariants: for example, *Satureja subspicata* subsp. *liburnica* and *S. subspicata* subsp. *subspicata* or *Satureja montana* subsp. *variegata* and *S. montana* subsp. *montana* (Šilić 1979). Moreover, the biogeographical importance of the Istrian Peninsula and the Kvarner area is emphasized by a number of endemic plant taxa with Mediterranean or (sub)Mediterranean distribution characteristics (e.g. *Asperula borbasiana*, *A. woloszcakii*, *Campanula fenestrellata* subsp. *istriaca*, *C. tommasinii, Centaurea dalmatica*, *Aurinia leucadea* subsp. *media*, *Moehringia tommasinii*). This further suggests that these areas acted as possible refugia during climatically unfavourable periods of the Quaternary. In a recent study on areas of endemism of the Mexican transition zone (Pinilla-Buitrago *et al.* 2018), the authors determined that such areas can experience changes through time as a response to climate, whereas others remain stable. However, in spite of some information from geomorphology, palynology and palaeoclimatology, the appearance of the eastern Adriatic coast and the Balkan Peninsula in general during the last glacial period is not clear. Due to the lower sea level, northern and central areas of the Adriatic basin were land masses (Correggiari *et al.* 1996). Even present-day coastal areas of the Dinaric Alps were affected by glaciations that reached the lowlands in the northernmost areas (Marjanac and Marjanac 2004; Menković *et al.* 2004; Hughes *et al.* 2010; Žebre *et al.* 2016). However, compared with inner areas of the Balkan Peninsula, paleoclimatic studies have suggested warmer and wetter conditions in the Adriatic basin (Kuhlemann *et al.* 2008, 2009). Likewise, palynological studies have suggested presence of thermophytic taxa in the Adriatic basin during the coldest periods (Schmidt *et al.* 2000), while the inner areas of the Balkan Peninsula were dominated by dry, open habitats characterized by the Chenopodiaceae and *Artemisia* spp. (Eastwood 2004). As previously mentioned, climatic conditions in the SE Adriatic were probably substantially mitigated by the proximity of the sea. Based on the paleoclimatic models, our results suggested warmer and wetter conditions in the environmentally suitable areas (putative refugia) compared with the neighbouring areas. However, the NW Adriatic refugium showed significantly lower temperature and precipitation values when juxtaposed to the SE refugium. Geomorphological representation of the Kvarner region during the LGM suggested the long-term presence of water masses in the deepest depressions (Benac and Juračić 1998), which might have mitigated the local climate. On the other hand, studies have shown that glaciation events affected even the lowlands (Marjanac and Marjanac 2004). The two areas recognized in our study as environmentally favourable probably acted differently as refugia. While environmental conditions in the SE Adriatic were more stable and more or less widespread, favourable conditions were limited to geographically confined micro-locations in the NW part.

### Patterns of morphological plasticity as a response to range dynamics

When studying characteristics of individual populations, the knowledge of the variability between populations is crucial; in order to ensure this, a representative sample in terms of geographical coverage and number of examined individuals has to be provided (Hodgins and Barrett 2008; Schouppe *et al.* 2017). The present study is based on a robust sample of individuals per population in the entire distribution range of the species. The results suggest that the past environmental conditions, through changes in range dynamics, have clearly left traces on the morphological characters of *E. tenuifolius*. The spatial segregation of populations based on different morphological characters evidently followed the partitioning as proposed by the genetic and environmental data. The strongest splits between populations were observed in central part of the distribution range (central Dalmatia), followed by strong barriers which were predicted to separate populations from the eastern Istrian Peninsula and the Kvarner areas. In this area, major differences in characters between analysed populations were also observed. Specimens from one population on the island of Cres reached minimal values of bract and flower number in inflorescence and sizes of measured characters. On the other hand, specimens from the nearby Istrian Peninsula were characterized by the maximum values of measured characters. The distinctiveness of populations in the NW part of the range had already been observed by previous authors (Janchen 1910; Lakušić 1974). Despite the fact that the results of the spatial principal component analysis on the first axes suggested an increasing variability cline towards the SE, our analyses could not recognize such variability in the SE part of the distribution range. Distinctive morphs recognized by Lakušić (1974) in the dolomitic areas of central Herzegovina (Pop 22; Fig. 1 and **Supporting Information—Table S1**) were also not supported by the results of the present study.

In the geographical context floral traits may vary for several reasons (Herrera *et al.* 2006). First, changes in floral traits can appear as a response of phenotypic plasticity due to environmental changes. Organisms that survived unfavourable climate periods in refugia were subjected to somewhat frequent changes. In warmer periods, these organisms spread to newly available habitats. In such circumstances, phenotypic plasticity played an important role in survival and ongoing persistence (Ghalambor *et al.* 2007). Also, populations on range limits tend to occur in less favourable habitats characterized by higher climatic variations and in lower and more variable densities. The evolutionary importance of phenotypic plasticity on range boundaries and in areas of range expansion has already been demonstrated by several studies (Volis *et al.* 1998; Molina-Montenegro and Naya 2012; Araújo *et al.* 2013; Mathiasen and Premoli 2016). However, floral characters show less plasticity then vegetative characters as a direct response of changing environmental conditions (Caruso 2006).

Second, variability of floral traits can arise as a consequence from genetic drift. The phylogeography (Surina *et al.* 2011) of *E. tenuifolius* proved that the genetic diversity in more northern populations was depauperated and that rare alleles are less common; however, the overall genetic structure and the present distribution of haplotypes indicate that NW regions of the eastern Adriatic coast could have also served as an important area of long-term presence.

Lastly, geographical variations of floral traits may occur as a response to divergent natural selection. An adaptation to new environments leads to the evolution of new characters as a consequence of natural selection (Reznick and Ghalambor 2001). Since Darwin, it has been well established that diversification in animal-pollinated angiosperms is related to the divergence of floral traits, which is promoted by adaptations to different pollinators. When considering this on a geographical level, one should consider that local adaptive floral diversification is a result of contrasting pollination environments (Dilley *et al.* 2000; Patterson and Givnish 2004). Diversification driven through pollinators should be considered across wider spectra, starting with adaptation (microevolution) up to speciation (macroevolution; Van der Niet *et al.* 2014). However, there is a significant shortage of studies on intraspecific floral variation and its relation to geographic divergence in pollinators (Herrera *et al.* 2006).

Another aspect to consider is the diversification of floral traits as a result of hybrid introgression. In the case of *Edraianthus*, morphological variability should be associated with potential hybridization events, especially in the central and southern parts of the Dinaric Alps, where the diversity of *Edraianthus* taxa is the highest and sympatry frequent. Evidence of reticulate evolution could be observed in the phylogenetic analysis of the genus *Edraianthu*s (Stefanović *et al.* 2008). A well-studied example of a hybrid between *E. tenuifolius* and *E. wettsteinii* subsp. *lovcenicus* is represented by *E. x lakusicii* from Mt. Lovčen in Montenegro (Lakušić *et al.* 2009).

To properly understand the mechanisms of gene flow within and between populations, much more consideration should be given to the understanding of reproductive ecology. The plant–pollinator relationship was emphasized as crucial to understanding historic biogeographical patterns (Maubecin *et al.* 2016). Very little is currently known about the reproductive biology of *Edraianthus*. The genus *Edraianthus* represents an entomophilous taxa, which is most likely pollinated by solitary bees and bumblebees, as has been observed in *Campanula* species with similar floral morphology (Blionis and Vokou 2001). However, in order to fully interpret intricate biogeographical patterns, the necessity of a better understanding of these relationships should continuously be emphasized.

## Conclusion

The present study has contributed to a deeper understanding of biodiversity patterns in the western Balkans. The validity of the ‘refugia within refugia’ model using an alternative methodological approach with respect to the well-established phylogeographical analysis was tested. The results suggested congruent biogeographical patterns to the already known phylogeographical structure. Hindcasts to LGM conditions suggested two separated areas of environmental suitability, one on the present-day N Adriatic coast of Croatia (Istrian Peninsula, Kvarner) and another on the present-day SE Adriatic coast (S Dalmatia, Montenegro and N Albania). Morphological variability suggested a similar pattern, where southern populations are separated from northern ones by a major split in the central part of its distribution range. However, detecting areas that represents potential ice age refugia is just the beginning of understanding biodiversity processes on a geographical level. Further efforts are needed to address the mechanisms that have led to present day-diversity in terms of environmental changes and ecological processes through recent Earth history.

## Sources of Funding

Funding by the bilateral project between Slovenia and Bosnia and Herzegovina BI-BA/14-15-026, the research programme ARRS P3-0384 and UP FAMNIT is gratefully acknowledged.

## Contributions by the Authors

B.S. and P.G. designed the study, P.G., B.S., T.R. and D.L. gained data on the distribution and collected the samples, P.G. and M.T. analysed the data, P.G. and B.S. wrote the first draft of the paper, M.T., V.B.G., T.R. and D.L. read and provided further comments.

## Conflict of Interest

None declared.

## Supporting Information

The following additional information is available in the online version of this article:

**Table S1.** Sampled populations (Pop) of *Edraianthus tenuifolius.*

**Table S2.** Different sets of variables evaluated for further model building based on AUC and AIC values.

**Table S3.** Comparison of three areas (central, northwestern (NW) and southeastern (SE)) according to minimum (min), average (avg) and maximum (max) values of selected bioclimatic variables, calculated as average values across three global circulation models (MIROC, CCSM and MPI-ESM-P).

**Table S4.** Comparison of three areas (intermediate, northwestern (NW) and southeastern (SE)) according to selected bioclimatic variables.

**Table S5.** Morphological characters of the *Edraianthus tenuifolius* inflorescence measured in this study.

**Table S6.** Statistical measures to assess the performance of the Kriging interpolation of morphological characters of the *Edraianthus tenuifolius* inflorescence.

**Fig. S1.** Visualization of subdivision of *Edraianthus tenuifolius* populations based on the results of this study using geostatistics (ISOCLUSTER) and Barrier (Barrier 1 (two groups) and Barrier 3 (four groups)).

Supporting Information
