# Supplementary material for: Understanding biogeographical patterns in the western Balkan Peninsula using environmental niche modelling and geostatistics in polymorphic Edraianthus tenuifolius
Source: AoB Plants. 2018 Oct 11;10(6):ply064. doi: 10.1093/aobpla/ply064 (PMC6220721; doi:10.1093/aobpla/ply064)
Supplement: Supporting Information [file ply064_suppl_supporting_information.docx]

[ **Supporting Information** ]

Table S1. Sampled populations (Pop) of *Edraianthus tenuifolius* (HRV: Croatia, BIH: Bosnia and Herzegovina, MNE: Montenegro). Structure, Beast and Haplotypes refer to the affiliation to a particular genetic cluster (here identified as geographic group) based on the results of AFLP and plastid DNA variation published in Surina *et al*. 2011.

| **Pop** | **Country** | **Region** | **Locality** | **Acronym** | **Elevation (m)** | **Latitude** | **Longitude** | **N° samples** | **Structure** | **Beast** | | **Haplotypes** |
| --- | --- | --- | --- | --- | --- | --- | --- | --- | --- | --- | --- | --- |
| 1 | HRV | Istrian Peninsula | Plomin, Sisol hill | PLOM | 560 | 45.1469° | 14.1843° | 20 | 1 | 1 | 1 | |
| 2 | HRV | Liburnian Karst, | Kvarner, Cres island, Sis hill | SIS | 390 | 45.0618° | 14.3618° | 20 | 1 | 1 | 1 | |
| 3 | HRV | Liburnian Karst, | Kvarner, Cres island, Belej | BELE | 123 | 44.7523° | 14.4280° | 20 | 1 | 1 | 1 | |
| 4 | HRV | Liburnian Karst, | Kvarner, Krk island, Omišalj, in the proximity of the Krk bridge | KRK | 70 | 45.2418° | 14.5614° | 20 | 1 | 1 | 1 | |
| 5 | HRV | Liburnian Karst, | Kvarner, Krk island, Baška, Hlam hill | HLAM | 365 | 44.9820° | 14.7600° | 20 | 1 | 1 | 1 | |
| 6 | HRV | Liburnian Karst | Grobnik, Grobničko polje | GRPO | 305 | 45.3785° | 14.5126° | 17 | 1 | 1 | 1 | |
| 7 | HRV | Liburnian Karst | Kvarner, Jadranovo | JADR | 150 | 45.2371° | 14.6196° | 20 | 1 | 1 | 1 | |
| 8 | HRV | Liburnian Karst | Kvarner, Sibinj, Tomišina Draga valley | TODR | 31 | 45.0549° | 14.8729° | 20 | 1 | 2 | 1 | |
| 9 | HRV | Liburnian Karst | Senj, village Matešiči | SENJ | 250 | 44.9704° | 14.9631° | 20 | 1 | 1 | 1 | |
| 10 | HRV | Velebit Mts. | Sveti Juraj, village Žrnovnica | SVJU | 57 | 44.9019° | 14.9125° | 20 | 1 | 1 | 1 | |
| 11 | HRV | Velebit Mts. | Karlobag, village Sušanj Cesarički | VELE | 610 | 44.5254° | 15.1183° | 21 | 1 | 1 | 1 | |
| 12 | BIH | Western Bosnia | Drvar, Trniča brijeg above Grubor | DRVA | 840 | 44.3307° | 16.3810° | 20 | 2 | 1 | 1 | |
| 13 | BIH | Western Herzegovina | Tomislavgrad, towards Šujica | ŠUJI | 1014 | 43.7944° | 17.2176° | 20 | 2 | 1 | 1 | |
| 14 | HRV | Northern Dalmatia | Pađene, towards Otrić | PAĐE | 352 | 44.1451° | 16.0643° | 20 | 2 | 1 | 1 | |
| 15 | HRV | Northern Dalmatia | Kijevo | KIJE | 557 | 44.0055° | 16.3236° | 20 | 2 | 1 | 1 | |
| 16 | HRV | Northern Dalmatia | Karin | KARI | 51 | 44.1263° | 15.6362° | 20 | 2 | 1 | 1 | |
| 17 | HRV | Northern Dalmatia | Zadar, Nadin | NADI | 177 | 44.0615° | 15.5325° | 8 | 2 | 1 | 1 | |
| 18 | HRV | Central Dalmatia | Murter island | MURT | 62 | 43.7980° | 15.6153° | 36 | 2 | 1 | 1 | |
| 19 | HRV | Central Dalmatia | Šibenik, Kamenar hill | ŠIBE | 250 | 43.7374° | 15.9151° | 20 | 2 | 1 | 1 | |
| 20 | HRV | Central Dalmatia | Omiš, Vrisovci | OMIŠ | 183 | 43.4244° | 16.7188° | 20 | 2 | 1 | 1 | |
| 21 | HRV | Central Dalmatia | Biokovo Mts., above Tučepi | BIOK | 560 | 43.2584° | 17.0899° | 11 | 2 | 1 | 1 | |
| 22 | BIH | Herzegovina | Konjic | KONJ | 330 | 43.6587° | 17.9622° | 21 | 3 | 1 | 2 | |
| 23 | BIH | Herzegovina | Jablanica | JABL | 195 | 43.6198° | 17.7443° | 17 | 3 | 1 | 2 | |
| 24 | BIH | Herzegovina | Mostar | MOST | 60 | 43.3571° | 17.8044° | 20 | 3 | 1 | 2 | |
| 25 | BIH | Herzegovina | Dubovica, between Blagaj and Nevesinje | NBLNE | 710 | 43.2508° | 17.9566° | 5 | 3 | 1 | 2 | |
| 26 | BIH | Herzegovina | Rilja, between Nevesinje and Gacko | RILJ | 917 | 43.2425° | 18.3244° | 20 | 3 | 1 | 2 | |
| 27 | HRV | Southern Dalmatia | Pelješac Peninsula, Pijavičino | PIJA | 360 | 42.9402° | 17.3756° | 20 | 3 | 3 | 2 | |
| 28 | HRV | Southern Dalmatia | Pelješac Peninsula, Putniković | PELJ | 160 | 42.8752° | 17.5202° | 20 | 3 | 3 | 2 | |
| 29 | HRV | Southern Dalmatia | Dubrovnik, Srđ hill | SRĐ | 282 | 42.6443° | 18.1218° | 20 | 3 | 1 | 2 | |
| 30 | BIH | Herzegovina | Trebinje | TREB | 355 | 42.7227° | 18.3453° | 20 | 3 | 1 | 2 | |
| 31 | MNE | Central Montenegro | Morača canyon, Potoci | MORA | 120 | 42.5394° | 19.3339° | 8 | 3 | 4 | 2 | |
| 32 | MNE | Northern Montenegro | Komarnica canyon, Čeoci | KOMA | 980 | 43.0047° | 19.0616° | 13 | 3 | 4 | 2 | |
| 33 | MNE | Southern Montenegro | Virpazar | VIRP | 112 | 42.2349° | 19.1057° | 36 | 3 | 5 | 2 | |
| 34 | MNE | Southern Montenegro | Podgorica, Cijevna valley | CIJE | 115 | 42.3986° | 19.3764° | 20 | 3 | 5 | 2 | |
| 35 | MNE | Southern Montenegro | Mt. Rumija, Kravari | RUMI | 471 | 42.0643° | 19.3741° | 12 | 3 | 5 | 2 | |

Table S2: Different sets of variables evaluated for further model building based on AUC and AIC values. For the chosen set of variables different *regularization multiplier* were evaluated based on best model performance (AIC). The chosen set is indicated in bold.

| Variables | Bio2 | Bio3 | | Bio4 | Bio7 | Bio8 | Bio9 | Bio12 | | Bio13 | Bio15 | Bio17 | AUC training | | AUC test | AIC | AICc | |
| --- | --- | --- | --- | --- | --- | --- | --- | --- | --- | --- | --- | --- | --- | --- | --- | --- | --- | --- |
| Model |  |  |  |  |  |  |  |  |  |  |  |  |  |  |  |  |  |  |
| 1 |  | + | | + | + | + | + | + | |  |  | + | 0.9945 | | 0.9174 | 3701.20 | 5501.77 | |
| 2 | + |  | | + |  | + | + |  | | + |  |  | 0.9945 | | 0.9110 | 3726.24 | 5007.13 | |
| 3 |  | + | |  | + | + |  |  | | + | + |  | 0.9922 | | 0.9279 | 3604.90 | 3624.09 | |
| 4 | + |  | | + |  | + | + | + | |  |  |  | 0.9945 | | 0.9164 | 3733.39 | 5240.89 | |
| **5** | **+** |  | |  | **+** | **+** | **+** |  | | **+** |  | **+** | **0.9523** | | **0.9327** | **3578.25** | **3614.89** | |
| Model 5 | | | Regularization multiplier | | | | | | AIC | | | | | AICc | | | |  |
|  |  |  | 1 | | | | | | 3585.94 | | | | | 3626.59 | | | |  |
|  |  |  | **5** | | | | | | **3624.70** | | | | | **3626.18** | | | |  |
|  |  |  | 10 | | | | | | 3648.67 | | | | | 3649.42 | | | |  |
|  |  |  | 20 | | | | | | 3660.31 | | | | | 3660.87 | | | |  |

Table S3. Comparison of three areas (central, northwestern – NW and southeastern – SE) according to minimum (min), average (avg) and maximum (max) values of selected bioclimatic variables, calculated as average values across three global circulation models (MIROC, CCSM and MPI-ESM-P): BIO1 - annual mean temperature, BIO5 - max temperature of warmest month, BIO6 - min temperature of coldest month, BIO12 - annual precipitation, BIO16 - precipitation of wettest quarter and BIO 17 - precipitation of driest quarter.

|  | **Central area** | | | **NW area** | | | **SE area** | | |
| --- | --- | --- | --- | --- | --- | --- | --- | --- | --- |
|  | **min** | **avg** | **max** | **min** | **avg** | **max** | **min** | **avg** | **max** |
| **BIO1** | -4.7 | 4.0 | 12.5 | -3.0 | 6.0 | 8.5 | 1.1 | 9.7 | 12.2 |
| **BIO5** | 10.7 | 20.7 | 28.3 | 13.8 | 22.5 | 25.3 | 15.5 | 20.0 | 27.9 |
| **BIO6** | -19.7 | -10.4 | 1.2 | -17.7 | -8.5 | -5.8 | -11.0 | -3.3 | 0.0 |
| **BIO12** | 846.7 | 1228.1 | 1921.3 | 971.0 | 1193.1 | 1449.7 | 1130.3 | 1469.5 | 1921.3 |
| **BIO16** | 296.0 | 425.5 | 749.3 | 323.7 | 402.1 | 494.0 | 405.0 | 560.8 | 749.3 |
| **BIO17** | 85.0 | 207.2 | 304.0 | 175.7 | 226.8 | 302.3 | 118.3 | 156.8 | 218.3 |

Table S4. Comparison of three areas (intermediate, northwestern – NW and southeastern – SE) according to selected bioclimatic variables: BIO1 - annual mean temperature, BIO5 - maximal temperature of warmest month, BIO6 - minimal temperature of coldest month, BIO12 - annual precipitation, BIO16 - precipitation of wettest quarter and BIO 17 - precipitation of driest quarter. Statistically significant differences (p < 0.05) are indicated with an asterisk (*).

| **Paleoclimatic model** | **Bioclim variable** | **NW vs. SE** | **NW vs. intermediate area** | **SE vs intermediate area** |
| --- | --- | --- | --- | --- |
| MPI –ESM-P | BIO1 | * | * | * |
|  | BIO5 | * | * | * |
|  | BIO6 | * | * | * |
|  | BIO12 | * | * | * |
|  | BIO16 | * | * | * |
|  | BIO17 | * | / | * |
| CCSM | BIO1 | * | * | * |
|  | BIO5 | * | * | * |
|  | BIO6 | * | / | * |
|  | BIO12 | * | * | * |
|  | BIO16 | * | * | * |
|  | BIO17 | * | * | * |
| MIROC | BIO1 | * | * | * |
|  | BIO5 | * | * | * |
|  | BIO6 | / | * | * |
|  | BIO12 | * | * | * |
|  | BIO16 | * | / | * |
|  | BIO17 | * | * | * |

Table S5. Morphological characters of the *Edraianthus tenuifolius* inflorescence measured in this study.

| **Characters** | | **Acronym** |
| --- | --- | --- |
| Number of bracts |  | BR |
| Number of flowers |  | FL |
| Outer bract | Maximal width | MW1 |
|  | Width in the upper quarter | MUQ1 |
|  | Total length | TL1 |
|  | Distance between widest point and the bract base | DBB1 |
|  | Length of the bract base | LBB1 |
| Central bract | Maximal width | MW2 |
|  | Width in the upper quarter | MUQ2 |
|  | Total length | TL2 |
|  | Distance between widest point and the bract base | DBB2 |
|  | Length of the bract base | LBB2 |
| Inner bract | Maximal width | MW3 |
|  | Width in the upper quarter | MUQ3 |
|  | Total length | TL3 |
|  | Distance between widest point and the bract base | DBB3 |
|  | Length of the bract base | LBB3 |
| Calyx (central flower) | Diameter | CaD |
|  | Calyx lobe base width | CaLBW |
|  | Calyx lobe width in the upper quarter | CaLWUQ |
|  | Calyx lobe total length | CaLTL |
| Corolla (central flower) | Maximal width | CoMW |
|  | Width of the lobe base | CoWLL |
|  | Total height | CoTH |
|  | Height of the lobe | CoHT |
| Style (central flower) | Length | SL |
| Anther (central flower) | Length | AL |
|  | Filament length | AFL |
|  | Length of filament base | ALFB |
| Calyx 2 (lateral flower) | Diameter | CaD2 |
|  | Calyx lobe base width | CaLBW2 |
|  | Calyx lobe width in the upper quarter | CaLWUQ2 |
|  | Calyx lobe total length | CaLTL2 |
| Corolla 2 (lateral flower) | Maximal width | CoMW2 |
|  | Width of the lobe base | CoWLL2 |
|  | Total height | CoTH2 |
|  | Height of the lobe | CoHT2 |
| Style 2 (lateral flower) | Length | SL2 |
| Anther 2 (lateral flower) | Length | AL2 |
|  | Filament length | AFL2 |
|  | Length of filament base | ALFB2 |

Table S6. Statistical measures to assess the performance of the Kriging interpolation of morphological characters of the *Edraianthus tenuifolius* inflorescence

| Character acronym | Mean | Root mean square | Mean standardized | Root-mean-square standardized | Average standard error |
| --- | --- | --- | --- | --- | --- |
| BR | -0.0529 | 1.4759 | -0.0685 | 1.1556 | 1.3202 |
| FL | -0.0281 | 1.7639 | -0.0171 | 1.0053 | 1.7630 |
| MW1 | -0.0458 | 1.1354 | -0.0314 | 1.1004 | 1.0141 |
| MUQ1 | -0.0015 | 0.2324 | -0.0069 | 0.9648 | 0.2417 |
| TL1 | -0.5234 | 8.0175 | -0.0480 | 0.9237 | 8.6832 |
| DBB1 | -0.0169 | 0.2978 | -0.0590 | 1.0154 | 0.2946 |
| LBB1 | -0.0350 | 0.8830 | -0.0357 | 1.0267 | 0.8596 |
| MW2 | -0.0598 | 1.3672 | -0.0306 | 1.1497 | 1.1965 |
| MUQ2 | -0.0026 | 0.1831 | -0.0131 | 0.9803 | 0.1868 |
| TL2 | -0.2633 | 5.8360 | -0.0442 | 1.0098 | 5.7919 |
| DBB2 | -0.0386 | 0.8423 | -0.0397 | 1.0773 | 0.7926 |
| LBB2 | -0.0742 | 1.3975 | -0.0475 | 1.0249 | 1.3516 |
| MW3 | -0.0132 | 0.6214 | -0.0172 | 1.0896 | 0.5662 |
| MUQ3 | -0.0029 | 0.1961 | -0.0127 | 1.2132 | 0.1585 |
| TL3 | -0.1356 | 3.7346 | -0.0398 | 1.0315 | 3.5797 |
| DBB3 | -0.0393 | 1.2931 | -0.0232 | 0.9735 | 1.3384 |
| LBB3 | -0.0900 | 1.7895 | -0.0495 | 1.0211 | 1.7459 |
| CaD | -0.0309 | 0.5069 | -0.0591 | 1.0106 | 0.5000 |
| CaLBW | -0.0152 | 0.2508 | -0.0563 | 0.9452 | 0.2652 |
| CaLWQ | -0.0005 | 0.1131 | -0.0040 | 1.0490 | 0.1074 |
| CaLTL | -0.0447 | 1.3823 | -0.0347 | 0.9735 | 1.4055 |
| CoMW | 0.0136 | 5.7548 | 0.0019 | 1.0729 | 5.2065 |
| CoWLL | -0.0353 | 0.6995 | -0.0454 | 0.9403 | 0.7425 |
| CoTH | -0.1349 | 3.8636 | -0.0308 | 0.9216 | 4.1369 |
| CoHT | -0.0446 | 1.6759 | -0.0225 | 0.9208 | 1.8301 |
| SL | -0.0818 | 2.4612 | -0.0309 | 0.9769 | 2.4933 |
| AL | -0.0636 | 1.1284 | -0.0558 | 0.9724 | 1.1344 |
| AFL | -0.0016 | 0.1083 | -0.0171 | 1.0519 | 0.1028 |
| ALFB | -0.0271 | 0.3916 | -0.0612 | 0.9425 | 0.4071 |
| CaD2 | -0.0068 | 1.3516 | -0.0062 | 1.1132 | 1.2018 |
| CaLBW2 | 0.0005 | 0.6477 | 0.0001 | 1.0732 | 0.5993 |
| CaLWQ2 | 0.0001 | 0.2215 | -0.0009 | 1.0337 | 0.2131 |
| CaLTL2 | -0.0089 | 2.6611 | -0.0044 | 1.0911 | 2.4230 |
| CoMW2 | 0.0139 | 7.5996 | 0.0013 | 1.0585 | 7.1382 |
| CoWLL2 | 0.0019 | 1.5463 | 0.0009 | 1.0586 | 1.4520 |
| CoTH2 | -0.0246 | 6.8530 | -0.0043 | 1.0821 | 6.2909 |
| CoHT2 | 0.0191 | 2.9194 | 0.0066 | 1.0498 | 2.7685 |
| SL2 | -0.0348 | 5.3303 | -0.0073 | 1.1101 | 4.7596 |
| AL2 | -0.0216 | 2.7581 | -0.0091 | 1.1267 | 2.4236 |
| AFL2 | -0.0020 | 0.1875 | -0.0112 | 1.0520 | 0.1775 |
| ALFB2 | -0.0077 | 0.7191 | -0.0117 | 1.1228 | 0.6337 |


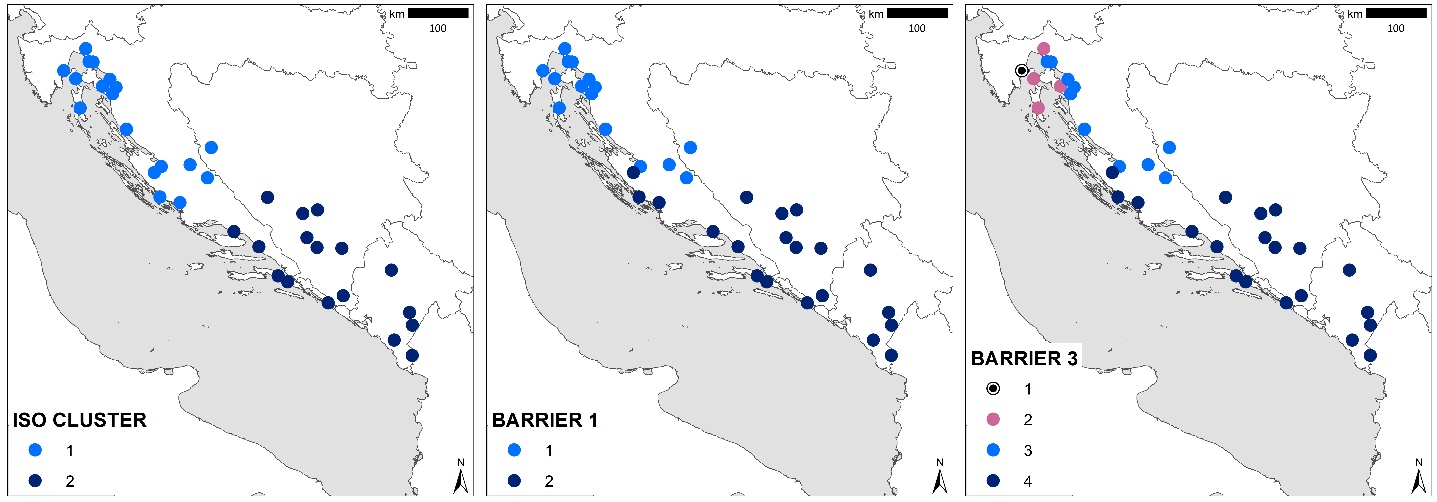


Fig. S1. Visualization of subdivision of *Edraianthus tenuifolius* populations based on the results of this study using geostatistics (ISOCLUSTER) and Barrier (Barrier 1 ‒ two groups and Barrier 3 ‒ four groups).
